# Non–Dioxin-Like Polychlorinated Biphenyls and Risk of Endometriosis

**DOI:** 10.1289/ehp.0901444

**Published:** 2010-04-27

**Authors:** Britton Trabert, Anneclaire J. De Roos, Stephen M. Schwartz, Ulrike Peters, Delia Scholes, Dana B. Barr, Victoria L. Holt

**Affiliations:** 1 University of Washington, Seattle, Washington, USA; 2 Fred Hutchinson Cancer Research Center, Seattle, Washington, USA; 3 Group Health Cooperative, Seattle, Washington, USA; 4 Centers for Disease Control and Prevention, Atlanta, Georgia, USA

**Keywords:** case–control, endometriosis, non–dioxin-like PCBs, population-based, risk factors

## Abstract

**Background:**

Endometriosis, a gynecologic disorder affecting 8–10% of reproductive-age women in the United States, is defined as the presence of endometrial tissue outside the uterus and is linked to pelvic pain and infertility. Environmental contaminants, including polychlorinated biphenyls (PCBs), are hypothesized to contribute to endometriosis risk through effects on steroid hormones.

**Objective:**

We evaluated serum concentrations of certain noncoplanar PCBs, which have no or only weak dioxin-like properties, as risk factors for endometriosis.

**Methods:**

In a case–control study of Group Health enrollees in western Washington State, 20 PCB congeners were measured in serum from surgically confirmed endometriosis cases that were newly diagnosed between 1996 and 2001 (*n* = 251) and from female controls matched for age and reference year (*n =* 538).

**Results:**

Summed and estrogenic PCB concentrations were not associated with endometriosis risk [summed: odds ratio (OR) = 1.3; 95% confidence interval (CI), 0.8–2.2; estrogenic: OR = 1.1; 95% CI, 0.8–1.4]. Although several congener-specific ORs were statistically above or below the null (PCB 170: third quartile vs. lowest: OR = 0.5; 95% CI, 0.3–0.9; PCB 196: third quartile vs. lowest: OR = 0.4; 95% CI, 0.2–0.7; PCB 201: second vs. lowest: OR = 0.5; 95% CI, 0.3–0.8; third quartile vs. lowest: OR = 0.4; 95% CI, 0.2–0.7), there were no overall consistent patterns of endometriosis risk.

**Conclusions:**

Taken in context with other North American studies, our findings suggest that noncoplanar PCB concentrations consistent within the range of exposure currently observed in western Washington State do not contribute meaningfully to endometriosis risk.

Endometriosis, defined as functioning endometrial glands and stroma outside the uterus, is characterized by internal bleeding, inflammation, scarring, and often infertility. The best population-based prevalence estimate suggests that 8–10% of reproductive-age women in the United States have endometriosis; thus, > 5.5 million women in North America are affected (Eskenazi and Warner 1997). The most common symptoms include intermenstrual spotting, heavy menstrual bleeding, painful cramps (dysmenorrhea), and painful intercourse (dyspareunia); women with long-term, severe endometriosis commonly are treated by hysterectomy and oophorectomy. Endometriosis develops mainly in reproductive-age women and typically regresses after menopause or oophorectomy, suggesting estrogen-dependent growth.

A growing body of literature suggests that environmental agents that alter endocrine function, either by altering hormone function or synthesis or by binding to estrogen or androgen receptors, may increase endometriosis risk (De Felip et al. 2004; Fierens et al. 2003; Heilier et al. 2007; Niskar et al. 2009; Nisolle et al. 1997; Pauwels et al. 2001; Reddy et al. 2006). Polychlorinated biphenyls (PCBs) are endocrine-disrupting agents widely used as dielectric fluids in transformers, capacitors, and coolants beginning in the early 1930s. Although PCB production was banned in the United States in 1976, PCBs persist in the air, water, and soil and have accumulated in the fatty tissue of fish, birds, and mammals worldwide. As a result, human exposure to PCBs occurs primarily through the consumption of animal and dairy products.

The role of human exposure to PCBs in the development of hormone-related diseases has been addressed in numerous epidemiologic studies since the early 1990s. The majority of studies of endometriosis to date have focused on dioxin-like PCBs (coplanar PCBs that induce biologic effects through binding to the aryl hydrocarbon receptor) and have generally found no association with endometriosis (De Felip et al. 2004; Fierens et al. 2003; Heilier et al. 2007; Niskar et al. 2009; Pauwels et al. 2001; Reddy et al. 2006). Studies exploring the association of non–dioxin-like PCB congeners (noncoplanar PCBs that have no or only weak dioxin-like toxicity) and endometriosis risk have been inconsistent, but they provide some evidence of association (Gerhard and Runnebaum 1992; Lebel et al. 1998; Louis et al. 2005; Pauwels et al. 2001; Porpora et al. 2006, 2009; Tsukino et al. 2005). The most recent of these, an Italian case–control study, reported an odds ratio (OR) as high as 4.9 for the association between PCB 153 exposure and endometriosis (Porpora et al. 2009). The existing studies of non–dioxin-like PCBs have been primarily small, infertility clinic- or hospital-based investigations that have enrolled as cases women undergoing ultrasound or surgical evaluation and found to have endometriosis. Controls in these studies were women who had also undergone ultrasound or surgical evaluation but were found to be free of endometriosis. To our knowledge no population-based study of non–dioxin-like PCB exposure and endometriosis risk has been conducted. We investigated the role of serum noncoplanar PCBs, primarily non–dioxin-like, as potential contributors to risk of endometriosis in a large, population-based, case–control study of women in Washington State.

## Materials and Methods

### Study population

Data for this study were collected as part of a previously described population-based case–control study of endometriosis, Women’s Risk of Endometriosis (WREN), conducted within Group Health (GH), a large mixed-model health care organization in the Pacific Northwest (Marino et al. 2008). Briefly, cases were female 18- to 49-year-old GH enrollees with an incident endometriosis diagnosis [*International Classification of Disease, 9th Revision* (ICD-9; World Health Organization 1977), diagnostic codes 617.0–617.5, 617.8, and 617.9, excluding individuals with ICD-9 code 617.0, uterine endometriosis, who actually had adenomyosis as determined by the pathology report] between 1 April 1996 and 31 March 2001. The date of each patient’s first GH visit for symptoms leading to the endometriosis diagnosis was determined through chart review and assigned as the reference date for cases. Population-based controls were randomly selected from a list of 18- to 49-year-old female GH enrollees during the same time period as the diagnoses of the cases. Controls were frequency matched to cases on 5-year age intervals, and each was assigned a reference date to correspond with the distribution of reference dates of the cases. Upon initial telephone eligibility screening, women who did not speak English or who reported a hysterectomy or bilateral oophorectomy were excluded from participation. All subjects provided informed consent and the GH Institutional Review Board approved all study protocols.

An interview ascertaining endometriosis risk factors was completed by 340 cases (73% of those eligible) and 741 controls (73% of those eligible). An interviewer asked questions from a structured questionnaire to ascertain information on exposures occurring prior to the reference date; the questionnaire also included questions regarding demographics, employment, prior medical conditions, menstrual history, pregnancy history, contraceptive methods, hormone use, tobacco and alcohol use, and family and personal history of endometriosis. Inpatient and outpatient medical records were reviewed for all consenting study participants; further, medical records of cases were abstracted for symptom type and severity as well as for endometriosis lesion characteristics. As a result of information captured in the interview, 12 cases and 14 controls with a prior history of surgically confirmed endometriosis but included in the original interview were excluded from analysis. We used a published case definition that emphasizes more serious disease. As a result, the case definition was further refined to include only those women with definite or probable endometriotic disease—disease causing structural or functional damage or substantial symptoms—or as defined by Holt and Weiss (2000). Under these criteria, definite endometriotic disease included ovarian endometriomas, pelvic endometriotic lesions > 5-mm deep, and pelvic endometriotic lesions with adhesions not attributable to other causes. Other endometriotic implants with at least one major endometriosis symptom (infertility, moderate to severe dysmenorrhea, dyspareunia, or pelvic pain) were classified as probable endometriotic disease. As a result, cases without surgical evidence of disease and asymptomatic cases with superficial or ambiguous pelvic lesions were excluded (*n* = 12), as well as three cases of extrapelvic scar endometriosis. After the interview, we asked all 1,022 study participants who were interviewed in person after funding for the PCB assays was obtained (93.5% of all cases and 94.6% of all controls) to donate 20 mL of blood; 78.7% of these cases and 76.5% of these controls agreed to participate in the blood draw. Measurement of PCB concentrations in serum was completed for 251 cases and 538 controls, the final analysis subset.

### PCB measurements

Serum samples from the blood draw of each study subject were processed at the Fred Hutchinson Cancer Research Center and stored in acid-washed glass vials at −20°C until shipment on dry ice to the laboratory in the Division of Laboratory Sciences, National Center for Environmental Health, [Centers for Disease Control and Prevention (CDC), Atlanta, GA]. All blood collection equipment and vials remained in their original packaging until use in the field. Wet-weight concentrations of 34 PCB congeners [IUPAC (International Union of Pure and Applied Chemistry) 18, 28, 44, 49, 52, 66, 74, 87, 99, 101, 118, 128, 138, 146, 149, 151, 153, 156, 157, 167, 170, 172, 177, 178, 180, 183, 187, 189, 194, 195, 196, 201, 206, and 209] were quantified in serum samples by accelerated solvent extraction with gel permeation chromatography purification followed by high-resolution gas chromatography/high-resolution mass spectrometry with isotope dilution quantification, based on methods previously published (Barr et al. 2003, 2006). The congeners evaluated are noncoplanar PCBs, and all are non–dioxin-like except PCBs 118 and 156, which cause only very weak dioxin-like toxicity (Van den Berg et al. 1998, 2006). The detection limit was 20 pg/g serum for congeners 18 and 28 and 5.0 pg/g serum for all other congeners.

Free cholesterol, total cholesterol, triglycerides, and phospholipids were measured in each serum sample using enzymatic methods (Roche Chemicals, Indianapolis, IN) (Phillips et al. 1989). Total lipid concentrations were calculated for each sample using published equations (Bernert et al. 2007; Phillips et al. 1989). We adjusted for intraindividual variations in serum wet-weight PCB concentrations resulting from fluctuations in serum lipid concentration by including a natural log-transformed total lipid variable as an independent variable in all statistical analyses.

### Statistical analysis

Information on number of samples with PCB measurements above the detection limit is provided in Supplemental Material, Table 1 (doi:10.1289/ehp.0901444). PCB congeners 87, 101, 128, 146, 149, 151, 157, 167, 172, 177, 178, 183, 189, and 195 were detected in < 75% of samples; thus, these congeners were excluded from further analyses presented here. Measurement reliability was assessed through comparison of 36 randomly selected quality control (QC) duplicates included in each run. The within-batch intraclass correlation coefficient (ICC) was calculated for each QC pair. The 20 congeners included in our statistical analyses showed high reliability, with a median ICC of 0.96 and all ICCs > 0.90.

We categorized wet-weight PCB concentrations according to the quartile distribution in controls. Values below the limit of detection were always included in the lowest exposure category, which served as the reference category. To assess a linear trend of the association between endometriosis risk and PCB concentration, we also fit models using the natural log-transformed continuous values for each PCB congener. Values below the limit of detection were set to missing for the log-linear models.

In addition to separately analyzing individual PCB congeners, we created two summary PCB variables. The sum of the PCB congeners (∑PCBs) in our analysis was computed by first converting the wet-weight PCB measure (picograms per gram serum) to moles per gram serum and then summing across the individual PCB congeners. Conversion to moles per gram was accomplished by dividing the wet-weight PCB value by the molecular weight for each congener. A second summary variable, estrogenic PCBs, was formulated by summing the molar concentrations of PCB congeners 18, 44, 49, 66, 74, and 99, which have been observed as having estrogenic potency using an assay based on *in vitro* estrogen-dependent proliferation of MCF-7 cells (DeCastro et al. 2006). ∑PCBs and estrogenic PCB concentrations were categorized into quartiles based on the distribution in controls, with the lowest category serving as the reference. To enable summing of PCB congeners for the entire study population, values below the detection limit were assigned a value of the detection limit divided by the square root of 2 for each PCB (Hornung and Reed 1990; Vo et al. 2008). To evaluate a linear trend, continuous models were also fit using the log-transformed imputed continuous value for each summary measure.

ORs and 95% confidence intervals (CIs) for the risk of endometriosis in association with serum PCB concentrations were estimated using unconditional logistic regression. We analyzed variables for each PCB congener (as quartile categories or the log-transformed continuous variable) or ∑PCB metric in separate models. All analyses were adjusted for the frequency-matching variables (5-year age group and year of enrollment), natural log-transformed total lipid value as a continuous variable, and the following confounders based on a ≥ 10% change in the beta coefficient for at least one quartile of ∑PCBs: alcohol (current, former, never use), income (< $35,000, $35,000–$69,999, > $70,000; $US) and quartile of serum *p,p*′-dichlorodiphenyl dichloroethylene (DDE; nanograms per liter). DDE was included as an *a priori* potential confounding factor because it was previously reported to be associated with endometriosis (Porpora et al. 2009), and DDE was modestly associated with endometriosis in our study. It was retained in the final model because it met our criteria for a model-based confounder. Other potential confounders considered in the models included race (Caucasian, African American, Asian American, other), education (< 12 years, 12 years, > 12 years), body mass index (BMI; < 25.0 kg/m^2^, 25.0–29.9 kg/m^2^, ≥ 30.0 kg/m^2^), physical activity (any vs. none), age at menarche (< 12 years, 12–13 years, > 13 years), first-degree family history of endometriosis (mother or full sister), history of breast-feeding (nulliparous, did not breast-feed, breast-fed ≤ 1 month, breast-fed > 1 month), smoking (current, former, never use), and marijuana use (current, former, never); however, these covariates did not satisfy our definition of a model-based confounding factor and were not included in our final models.

Because the inability to become pregnant may be a consequence of endometriosis rather than a risk factor for the disease, we did not include parity as a potential confounding factor in our analyses. However, we did evaluate concentrations of ∑PCBs and estrogenic PCB as risk factors for endometriosis within strata of nulliparous and parous women.

Endometriosis is a heterogeneous disease entity, and ovarian and nonovarian endometriosis may have different etiologies. Therefore, in one subanalysis, we evaluated the association between PCB congeners and ovarian endometriosis and nonovarian pelvic endometriosis, separately. We also considered separately cases who reported seeking care only for reasons other than infertility, because women who seek treatment for infertility may have endometriosis discovered incidentally as part of the diagnostic process rather than because of symptomatic disease. Finally, because of the possibility that there may be undiagnosed symptomatic cases in our population-based control group, we also conducted a subanalysis comparing endometriosis cases only with asymptomatic controls.

Data analyses were performed using Stata software (Version 10.1 for Windows; (StataCorp, College Station, TX). The threshold for significance was set at *p* ≤ 0.05. The study protocol was reviewed and approved by the Institutional Review Boards at GH and Fred Hutchinson Cancer Research Center.

## Results

The distributions of selected demographic and health characteristics for the case and control women with PCB laboratory measurements are provided in Table 1. Cases and controls were similar with regard to race, education, and BMI. A higher percentage of cases than controls were current alcohol users (chi-square *p*-value = 0.02). The distribution of demographic and health characteristics were similar for study subjects who did and did not provide blood samples (results not shown). The population median wet-weight serum PCB concentration and the minimum and maximum detected concentrations of each PCB congener are shown in Supplemental Material, Table 1 (doi:10.1289/ehp.0901444). For those congeners with > 25% of samples below the limit of detection (PCB congeners 87, 101, 128, 149, 151, 157, 167, 172, 177, 178, 183, and 189), the percentage of quantified samples did not differ by disease status, nor were the quantified PCB congeners associated with endometriosis in analyses of categorized PCB concentration with observations below the detection limit included in the reference category (results not shown).

Higher quartiles of ∑PCB concentrations and estrogenic PCB concentrations were not associated with endometriosis risk (Table 2). In congener-specific analyses, modestly elevated—albeit not statistically significant—ORs were observed for PCBs 44, 49, 118, and 138 in some quartiles (maximum OR = 1.5). We observed inverse associations of PCB 170, PCB 196, and PCB 201 concentrations with endometriosis risk in some quartiles; however, there were no overall consistent patterns of endometriosis risk with these congeners. Using the natural log-transformed continuous PCB measures, we found no statistically significant log-linear associations with endometriosis risk for any of the congeners assessed.

Stratifying on parity, neither ∑PCB nor estrogenic PCB concentrations were associated with endometriosis risk, similar to our analysis of all women combined (Table 3). Similarly, individual PCB congeners were not associated with endometriosis risk when stratified by parity (results not shown). Our results were not substantially different when restricted to ovarian endometriosis cases only, nonovarian pelvic endometriosis cases only, or non–infertility-related case status (results not shown). In post hoc analyses excluding DDE as a potential confounding factor (because of potential collinearity with PCB), there was little difference in the overall interpretation of the results (results not shown). Compared with the results in Tables 2 and 3, the ORs were not increased in analyses restricted to asymptomatic controls, suggesting that the presence of undiagnosed symptomatic cases in our control group was unlikely to have affected our study (results not shown).

## Discussion

To our knowledge, this is the first population-based case–control study of the association between non–dioxin-like PCB concentrations and endometriosis. After controlling for age, reference year, income, alcohol consumption, serum lipids, and serum DDE, we observed statistically significant inverse associations for PCBs 170, 196, and 201 with endometriosis risk. Given the large number of statistical tests conducted, we would have expected at least three statistically significant associations; thus, these quartile associations are likely due to chance. Further, these associations have not been reported elsewhere, and they did not exhibit exposure–response relationships that would strengthen evidence for causation. The ORs calculated using quartiles of ∑PCB and estrogenic PCB serum concentrations provided evidence against an association between non–dioxin-like PCBs and endometriosis, and the lack of association was consistent in both parous and nulliparous subgroups. Additionally, there were no consistent log- linear trends in the association between ∑PCB-, estrogenic PCB–, or congener-specific PCB levels and endometriosis risk.

Several studies have evaluated non–dioxin-like PCBs and endometriosis risk, with inconsistent results (Lebel et al. 1998; Louis et al. 2005; Pauwels et al. 2001; Porpora et al. 2006; Reddy et al. 2006; Tsukino et al. 2005). There was a suggestion of increased endometriosis risk with PCBs 138, 153, and 180 in a German study (Gerhard and Runnebaum 1992) and in two studies conducted in Rome, Italy, with overlapping study populations (Porpora et al. 2006, 2009), findings that were not replicated in our study or in other studies (Lebel et al. 1998; Louis et al. 2005; Pauwels et al. 2001; Tsukino et al. 2005). In the study conducted in Germany, Gerhard and Runnebaum (1992) reported significantly higher mean serum concentrations of PCBs 138, 153, and 180 among women with endometriosis (*n =* 28) compared with women without endometriosis (*n =* 441). The authors evaluated PCB and endometriosis risk in a population of women attending a medical center at the University of Heidelberg for “hormone disturbances” and compared mean PCB concentrations in women with endometriosis and women without endometriosis. It is unclear whether the presence of endometriosis was laparoscopically or histologically confirmed, and it does not appear that the authors adjusted for serum lipids when making comparisons.

In the most recent and largest of the two Italian studies, Porpora et al. (2009) reported significantly elevated concentrations of PCBs 118, 138, 153, 170, and 180 in cases (*n =* 80) with laparoscopically diagnosed endometriosis compared with the controls (*n =* 78), nulliparous women with benign gynecologic conditions and no evidence of endometriosis. In contrast to that study, our study was not restricted to nulliparous women; however, we did evaluate our PCB–endometriosis associations stratified by parity, and our conclusions were not substantially different among nulliparous women. Our results may have differed because our control group was not restricted to women undergoing laparoscopy; rather, it was a random sample of reproductive-age women from the GH population. Furthermore, we included serum lipids as a covariate in our model rather than using lipid-standardized PCB concentrations, because the latter may be prone to bias, depending on the hypothesized underlying PCB–lipid disease association (Schisterman et al. 2005). Finally, differences between our results and the study by Porpora et al. (2009) may be due to geographic variability in PCB concentrations. The lipid-adjusted geometric mean PCB congener concentrations PCBs 138, 153, and 180 were almost twice as high in the control population from Rome than in our Washington State control population (e.g., the geometric mean concentration for PCB 153 was 61.8 ng/g lipid in the Porpora study, compared with 32.4 ng/g lipid among those detected in the present study), and it is possible that PCBs contribute to endometriosis risk only at higher concentrations than those observed in our study.

The remaining studies that evaluated non–dioxin-like PCBs and endometriosis risk have reported a lack of association between ∑PCB concentrations and endometriosis risk; although they have included PCBs 118, 138, 153, and 180, most have not reported congener- specific results, further limiting comparison among studies (Lebel et al. 1998; Louis et al. 2005; Pauwels et al. 2001; Tsukino et al. 2005). One pilot study conducted at an infertility clinic in Belgium (Pauwels et al. 2001) found no association of lipid-adjusted concentrations of PCB congeners 118, 138, 153, or 180 and endometriosis risk, in contrast to the German (Gerhard and Runnebaum 1992) and Italian studies (Porpora et al. 2006, 2009). The lipid-adjusted median values of these congeners were higher in the Belgian population (Pauwels et al. 2001) than than those from our study population (results not shown). However, the selection of infertile controls in the Belgian study may have masked an association, if present.

In a recent small U.S. study that adjusted for serum lipids as a covariate, as we did, Louis et al. (2005) reported no association between ∑PCB or estrogenic PCB concentrations and endometriosis risk. The study enrolled 84 consecutive women undergoing laparoscopies at two university-affiliated hospitals in Buffalo, New York, and compared endometriosis cases (*n =* 32) with women diagnosed with other gynecologic pathology or tubal sterilization and without endometriosis (*n =* 52).

The present study has several strengths, including its population-based design and large sample size. Cases and controls were all members of the same health maintenance organization, eliminating most issues pertaining to disparity of access to medical care. Chosen from the well-enumerated population of GH members, the race, income, and educational profile of the control population was similar to that of other female western Washington State residents (Saunders et al. 2008), and serum PCB concentrations were similar to the U.S. female population of reproductive age (CDC 2001). The availability of detailed questionnaire information enabled the adjustment for potential confounding effects. Furthermore, we conducted analyses of individual PCB congeners in addition to ∑PCBs and estrogenic PCBs to facilitate comparison with other studies.

To improve the sensitivity of our study, we used a well-defined set of criteria to evaluate the certainty of endometriosis diagnosis. Disease features and evidence were evaluated directly from medical records rather than relying on self-report. We excluded cases without surgical confirmation of endometriosis, as well as those women with a previous history of surgically confirmed endometriosis.

We selected as controls a random sample of women from the GH population rather than a group of women who had undergone surgical evaluation and been diagnosed with other gynecological conditions. We made this choice because of the possibility that women with other gynecological conditions, many of which are estrogen related, may not be representative of the population at risk in terms of their PCB exposure. If women had abnormally high PCB levels, the use of the control group could potentially mask any true association between PCB exposure and endometriosis.

One consequence of our choice was that some of our controls may have had undiagnosed endometriosis. The presence of undiagnosed, symptomatic endometriosis in the control group was likely to have been < 2%, resulting in a very small number of cases being misclassified as controls (Holt and Weiss 2000). Partly to address this issue, we limited our case group to women with definite or probable endometriotic disease, and it is unlikely that participants with this extent of disease were included in the control group. To further address the issue, in one subanalysis we excluded controls with endometriosis-type symptoms. In that analysis we found no change in our effect estimates, suggesting that the potential presence of undiagnosed cases in our control group had little impact on the results of the analyses we present here.

The postdiagnostic assessment of exposure levels is a limitation that is characteristic of all retrospective case–control studies of blood biomarkers and chronic disease. If PCB concentrations are affected by the disease, measured exposure levels may not be representative of past exposure occurring during a postulated period of causation. Weight change, which may affect PCB levels, is not a typical symptom of endometriosis onset; therefore, we do not consider this type of exposure misclassification to be likely. Although little information existed at the time of this study as to diets that would be effective in preventing disease recurrence, cases may nonetheless have modified their dietary habits after diagnosis. If cases decreased their meat or fat intake as a result of their endometriosis diagnosis, then the PCB concentrations in our case population may be underestimates of the true prediagnosis exposure levels. It is also possible that other unidentified behavioral changes may be made as a result of an endometriosis diagnosis; if these changes affect PCB levels, they may have impacted our study results. There is some evidence from animal studies that exposure to high PCB concentrations can affect growth of endometrial cells (Johnson et al. 1997; Rier et al. 1993); although we cannot rule out a minimal association in our data, it is likely that exposure to higher concentrations than those observed in the present study would be required to have an appreciable impact on endometriosis risk if a causal association truly does exist. Taken in context with results of other recent North American studies, our findings indicate that PCB concentrations consistent within the range of exposure currently observed in western Washington State do not contribute meaningfully to endometriosis risk. If patterns of decreasing PCB body burden continue in the general U.S. population as they have since the PCB ban in 1976, our results suggest that environmental PCB exposure is likely too low to play a measurable role in the etiology of endometriosis. Further evaluation in population-based studies conducted in occupational or environmental settings is needed to assess associations with higher PCB exposures.

## Figures and Tables

**Table 1:** Demographic and health characteristics of endometriosis cases (*n* = 251) and controls (*n* = 538), GH, 1996–2001.

Characteristic	Cases *n* (%)^a^	Controls *n* (%)^a^	*p*-Value
Age (years)			
18–24	20 (8.0)	44 (8.2)	
25–34	52 (20.7)	93 (17.3)	
35–44	121 (48.2)	277 (51.5)	
45–49	58 (23.1)	124 (23.0)	

Race			
Caucasian	207 (82.5)	444 (82.5)	
African American	8 (3.2)	23 (4.3)	
Asian American	13 (5.2)	36 (6.7)	
Other	23 (9.2)	34 (6.3)	0.38

Income (US$)			
< 35,000	75 (29.9)	146 (27.1)	
35,000–69,999	106 (42.2)	223 (41.4)	
≥ 70,000	61 (24.3)	153 (28.4)	0.56

Education (years)			
< 12	8 (3.2)	17 (3.2)	
12	44 (17.5)	96 (17.8)	
> 12	199 (79.3)	425 (79.0)	0.99

BMI (kg/m^2^)			
Underweight (< 18.5)	8 (3.2)	10 (1.9)	
Normal (18.5–24.9)	127 (50.6)	279 (51.9)	
Overweight (25–29.9)	62 (24.7)	140 (26.0)	
Obese (≥ 30)	54 (21.5)	105 (19.5)	0.61

Physical activity			
Any physical activity	194 (77.3)	429 (79.7)	
No physical activity	57 (22.7)	108 (20.1)	0.40

Cigarette smoking			
Current	51 (20.3)	89 (16.5)	
Former	57 (22.7)	124 (23.0)	
Never	143 (57.0)	325 (60.4)	0.42

Alcohol use			
Current	214 (50.8)	162 (44.0)	
Former	85 (20.2)	73 (19.8)	
Never	121 (28.7)	133 (36.1)	0.02

Parity			
Nulliparous	122 (48.6)	158 (29.4)	
Parous	129 (51.4)	379 (70.4)	0.01

History of breast-feeding among parous women			
Did not breast-feed	28 (21.7)	72 (19.1)	
≤ 1 month	11 (8.5)	18 (4.8)	
> 1 month	90 (69.8)	288 (75.9)	0.20

DDE quartile (ng/L)			
≤ 900	56 (22.3)	134 (24.9)	
901–1,575	70 (27.9)	135 (25.1)	
1,576–2,820	62 (24.7)	135 (25.1)	
> 2,820	63 (25.1)	134 (24.9)	0.58

Total DDE (ng/L) median (Q1, Q3)	1569.7 (947.5, 2825.3)	1574.5 (900.0, 2818.2)	

Total lipids (mg/dL) median (Q1, Q3)	679.6 (588.5, 814.3)	653.8 (567.3, 769.1)	

a Numbers may not sum to column total because of missing data.

**Table 2:** ORs (95% CIs) for association between wet-weight serum PCB congeners (modeled independently) and endometriosis, GH, 1996–2001.

	Reference	Quartile 2	Quartile 3	Quartile 4^a^	Log-linear^b^
∑PCBs	44/96^c^	45/98	42/99	57/95	
Adjusted	1.0	0.9 (0.5–1.7)	0.9 (0.5–1.6)	1.2 (0.6–2.3)	1.3 (0.8–2.2)

Estrogenic PCB^c^	58/116	47/117	57/116	59/116	
Adjusted	1.0	0.7 (0.4–1.1)	0.8 (0.5–1.3)	0.9 (0.5–1.4)	1.1 (0.8–1.4)

PCB 18	57/133	67/135	62/132	65/135	
Adjusted	1.0	1.1 (0.7–1.7)	1.0 (0.6–1.6)	1.0 (0.7–1.6)	1.0 (0.8–1.2)

PCB 28	57/133	71/137	59/134	64/134	
Adjusted	1.0	1.2 (0.8–1.8)	1.0 (0.6–1.5)	1.0 (0.6–1.6)	1.0 (0.8–1.2)

PCB 44	50/125	77/124	57/132	61/123	
Adjusted	1.0	1.5 (1.0–2.4)	1.0 (0.6–1.6)	1.2 (0.7–1.9)	1.0 (0.7–1.4)

PCB 49	49/133	71/125	44/125	67/126	
Adjusted	1.0	1.4 (0.9–2.2)	0.9 (0.5–1.4)	1.3 (0.8–2.1)	1.1 (0.8–1.5)

PCB 52	58/127	61/128	58/132	68/128	
Adjusted	1.0	1.0 (0.6–1.6)	0.9 (0.6–1.4)	1.2 (0.7–1.8)	0.9 (0.7–1.2)

PCB 66	58/134	73/131	57/129	59/132	
Adjusted	1.0	1.3 (0.8–2.0)	1.0 (0.6–1.6)	1.0 (0.6–1.5)	1.0 (0.8–1.3)

PCB 74	61/136	48/132	63/134	78/134	
Adjusted	1.0	0.8 (0.5–1.3)	1.0 (0.6–1.6)	1.2 (0.7–2.1)	1.2 (0.9–1.7)

PCB 99	51/130	71/130	58/128	65/131	
Adjusted	1.0	1.2 (0.7–1.9)	0.8 (0.5–1.5)	1.0 (0.6–1.8)	1.0 (0.7–1.3)

PCB 118	50/129	58/130	74/130	68/130	
Adjusted	1.0	1.2 (0.7–1.9)	1.4 (0.9–2.4)	1.3 (0.8–2.3)	1.0 (0.8–1.3)

PCB 138	52/129	72/131	52/131	69/128	
Adjusted	1.0	1.4 (0.8–2.2)	0.9 (0.5–1.7)	1.2 (0.7–2.3)	1.0 (0.7–1.4)

PCB 153	58/133	64/132	51/133	73/131	
Adjusted	1.0	1.1 (0.7–1.8)	0.8 (0.5–1.5)	1.2 (0.7–2.2)	1.1 (0.8–1.6)

PCB 156	57/122	55/121	39/100	80/143	
Adjusted	1.0	0.9 (0.6–1.6)	0.8 (0.5–1.5)	1.1 (0.6–2.0)	1.0 (0.7–1.4)

PCB 170	69/134	57/128	43/126	74/127	
Adjusted	1.0	0.8 (0.5–1.2)	0.5 (0.3–0.9)*	0.9 (0.5–1.5)	1.0 (0.7–1.4)

PCB 180	69/132	54/134	57/133	69/133	
Adjusted	1.0	0.7 (0.4–1.1)	0.7 (0.4–1.1)	0.8 (0.5–1.4)	0.9 (0.7–1.2)

PCB 187	59/121	57/119	53/125	66/117	
Adjusted	1.0	1.2 (0.7–2.0)	1.0 (0.5–1.7)	1.3 (0.7–2.4)	1.0 (0.7–1.4)

PCB 194	67/128	43/120	51/126	66/125	
Adjusted	1.0	0.7 (0.4–1.2)	0.7 (0.4–1.2)	0.9 (0.5–1.6)	1.0 (0.7–1.4)

PCB 196	61/122	56/126	40/132	74/123	
Adjusted	1.0	0.7 (0.4–1.2)	0.4 (0.2–0.7)*	0.9 (0.5–1.7)	1.0 (0.7–1.3)

PCB 201	70/120	41/125	44/126	73/126	
Adjusted	1.0	0.5 (0.3–0.8)*	0.4 (0.2–0.7)*	0.7 (0.4–1.3)	0.9 (0.7–1.3)

PCB 206	61/130	51/120	59/147	65/123	
Adjusted	1.0	0.8 (0.5–1.4)	0.7 (0.4–1.3)	1.0 (0.5–1.8)	0.9 (0.7–1.2)

PCB 209	66/114	54/121	58/133	51/123	
Adjusted	1.0	0.8 (0.5–1.3)	0.7 (0.4–1.2)	0.7 (0.4–1.2)	0.8 (0.6–1.1)

Values shown are number of cases/number of controls and ORs (95% CIs) adjusted for matching factors, log total serum lipids, income, alcohol consumption, and DDE exposure.

a Quartile cut points are provided in Supplemental Material, Table 1 (doi:10.1289/ehp.0901233).

b Data from the log-linear continuous model; OR is for a one-unit increase in the natural log-transformed wet-weight PCB (picograms per gram serum) concentration.

c Estrogenic PCBs include PCB congeners 18, 44, 49, 66, 74, 99.

* *p* < 0.05.

**Table 3:** ORs (95% CIs) for association of summed and estrogenic serum PCB congeners and endometriosis risk, GH, 1996–2001.

	Reference	Quartile 2	Quartile 3	Quartile 4	*p*-Value interaction
∑PCBs					
Nulliparous women	1.0	1.4 (0.5–3.8)	0.6 (0.2–1.6)	0.9 (0.3–2.6)	0.21
Parous women	1.0	0.7 (0.3–1.6)	0.9 (0.4–2.2)	1.2 (0.5–2.9)	
Estrogenic PCBs^a^					
Nulliparous women	1.0	1.4 (0.6–3.2)	1.1 (0.5–2.4)	0.8 (0.4–1.8)	0.16
Parous women	1.0	0.5 (0.2–0.9)	0.6 (0.3–1.2)	0.8 (0.4–1.5)	

Models were adjusted for matching factors, log-lipids, income, alcohol consumption, and DDE exposure.

a Estrogenic PCBs include PCB congeners 18, 44, 49, 66, 74, 99.
